# The Prevalence of Post-traumatic Stress Disorder Symptoms, Sleep Problems, and Psychological Distress Among COVID-19 Frontline Healthcare Workers in Taiwan

**DOI:** 10.3389/fpsyt.2021.705657

**Published:** 2021-07-12

**Authors:** Mei-Yun Lu, Daniel Kwasi Ahorsu, Shikha Kukreti, Carol Strong, Yi-Hsuan Lin, Yi-Jie Kuo, Yu-Pin Chen, Chung-Ying Lin, Po-Lin Chen, Nai-Ying Ko, Wen-Chien Ko

**Affiliations:** ^1^Department of Public Health, College of Medicine, National Cheng Kung University Hospital, National Cheng Kung University, Tainan, Taiwan; ^2^Center for Infection Control, National Cheng Kung University Hospital, Tainan, Taiwan; ^3^Department of Rehabilitation Sciences, Faculty of Health & Social Sciences, The Hong Kong Polytechnic University, Hung Hom, Hong Kong; ^4^Department of Nursing, College of Medicine, National Cheng Kung University, Tainan, Taiwan; ^5^Department of Orthopedic Surgery, Wan Fang Hospital, Taipei Medical University, Taipei, Taiwan; ^6^Department of Orthopedic Surgery, School of Medicine, College of Medicine, Taipei Medical University, Taipei, Taiwan; ^7^Institute of Allied Health Sciences, College of Medicine, National Cheng Kung University Hospital, National Cheng Kung University, Tainan, Taiwan; ^8^Department of Public Health, College of Medicine, National Cheng Kung University Hospital, National Cheng Kung University, Tainan, Taiwan; ^9^Department of Occupational Therapy, College of Medicine, National Cheng Kung University, Tainan, Taiwan; ^10^Department of Medicine, College of Medicine, National Cheng Kung University, Tainan, Taiwan; ^11^Department of Internal Medicine, Center for Infection Control, National Cheng Kung University Hospital, Tainan, Taiwan

**Keywords:** post-traumatic stress disorder, insomnia, psychological distress, healthcare workers, COVID-19, stigma

## Abstract

The adverse effect of COVID-19 pandemic among individuals has been very disturbing especially among healthcare workers. This study aims to examine the prevalence of post-traumatic stress disorder (PTSD) symptoms, sleep problems, and psychological distress among COVID-19 frontline healthcare workers in Taiwan. Hence, a total of 500 frontline healthcare workers were recruited to participate in this cross-sectional study. They responded to measures on fear of COVID-19, depression, anxiety, stress, insomnia, PTSD, perceived stigma, and self-stigma. The results indicated a prevalence rate of 15.4% for PTSD symptoms, 44.6% for insomnia, 25.6% for depressive symptoms, 30.6% for anxiety symptoms, and 23.4% for stress among the participants. There were significantly positive interrelationships between all these variables. Anxiety symptoms and fear of COVID-19 predicted PTSD whereas symptoms of anxiety, fear of COVID-19, and stress predicted insomnia. The prevalence rates of the psychological problems reveal a worrying view of mental health challenges among Taiwanese frontline healthcare workers. Anxiety symptoms and fear of COVID-19 are the common predictive factors of PTSD and sleep problems suggesting that mental healthcare services for them may help prevent future occurrence of psychological problems by allaying fears of healthcare workers. Therefore, there should be mental healthcare services for healthcare workers during the COVID-19 pandemic.

## Introduction

The coronavirus disease 2019 (COVID-19) pandemic has significantly altered our way of life (1–3), negatively affected our health (4–6) and debilitated economies worldwide (7, 8). During the study period (December 27, 2020), over 79.2 million people have contracted COVID-19, with fatalities around 1.7 million worldwide (9) and specifically, among Taiwanese, 785 people have contracted COVID-19 with 7 fatalities, 653 recovered, and 125 still hospitalised (10). With COVID-19 being a critical health issue, healthcare personnel especially those at the frontline face a daunting task of learning to convey appropriate information about COVID-19 to the population without inciting panic, protecting themselves from contracting the virus without compromising treatment efficacy, and dealing with other stressors that are associated with COVID-19 (11). Hence, frontline healthcare workers may have psychological challenges due to the stress involved with working in a COVID-19 environment daily. According to the transactional model of stress, stress may trigger predisposed illness in any individual without the use of appropriate coping strategies (12–14). Thus, poorly managed stressors may lead to psychological problems such as sleep problems, depression, and anxiety (14–18). Therefore, it may be prudent to examine the prevalence of psychological distress among frontline healthcare workers.

Also, during these life-saving activities, some healthcare workers contract COVID-19 with fatal outcome especially among doctors and nurses (19, 20). This may leave the surviving colleagues traumatised knowing that they may also contract COVID-19 which may lead to death. This trauma may further lead to stress-related disorders such as acute stress disorder (ASD) or post-traumatic stress disorder (PTSD) which may negatively affect their life. This may seriously impact their psychological well-being including their sleep as indicated by previous studies—insomnia, depression, anxiety, and stress (21, 22). Consequently, this may affect productivity due to the constant fear of contracting COVID-19 (17, 23). Additionally, due to the negative connotation of COVID-19, survivors of the disease suffer from stigma (from others—perceived stigma or self—self-stigma) with healthcare workers being one of the main victims (24–26). Unfortunately, this happens among healthcare workers themselves which may further lead to self-stigmatising attitudes. These attitudes are detrimental to the psychological well-being of the healthcare worker and may have cascade adverse effect on their work and social relationships (24–26). Getting enough information on attitude towards COVID-19 and its association with psychological outcomes may help in preventive measures and further allay public fears on COVID-19. Hence, this study aimed to examine the prevalence of post-traumatic stress disorder, sleep problems, psychological distress, and their correlates among healthcare workers. Apart from examining the prevalence rates of PTSD and psychological distress among the frontline healthcare workers, the study hypothesised that (1) there would be significant relationships between the variables used; (2) perceived stigma, depression, anxiety, stress, fear of COVID-19, and self-stigma would predict PTSD, and (3) perceived stigma, depression, anxiety, stress, fear of COVID-19, and self-stigma would predict insomnia.

## Method

### Participants and Procedure

This cross-sectional design study recruited 500 COVID-19 frontline healthcare workers who were available and willing to participate in this study at the National Cheng Kung University Hospital (NCKUH) in Tainan, Taiwan. The NCKUH is the largest medical centre in southern Taiwan and has more than 5,000 employees and more than 1,500 beds. The target participants (i.e., frontline healthcare workers across all departments in the NCKUH) were approached by the first author, who is a registered nurse in NCKUH, to obtain the study information. The first author clearly explained the study purpose to the target participants and provided a link with a QR code for those who are interested to participate in the study. Specifically, the healthcare workers who were interested in the study could freely log on to the link, which led them to a survey website (using the SurveyCake) for participation. Detailed information regarding the present study was also provided on the first page of the survey website and only when a participant hit the agree icon on the website could continue the survey. The study protocol has been approved by the Institutional Review Board of the National Cheng Kung University Hospital (NCKUH) in Tainan, Taiwan with the IRB number A-ER-109-149. The survey period was between September 24 and November 21, 2020.

### Measures

#### Fear of COVID-19

The healthcare workers' fear of coronavirus was assessed using FCV-19S developed by Ahorsu, Lin (27). FCV-19S is a seven-item self-report scale rated on a five-point Likert-type scale response format (strongly disagree = 1 to strongly agree = 5). The participants' responses are added together to generate the total score which ranges from 7 to 35. Hence, higher scores indicate greater fear of COVID-19. It has an acceptable internal consistency (Cronbach's α = 0.88). The Chinese version with linguistic validity was used for this study (28). The Cronbach's alpha coefficient is 0.87 for this study.

#### Depression, Anxiety, and Stress Scale-21

The healthcare workers' psychological distress was assessed using the DASS-21 developed by Lovibond and Lovibond (29). DASS-21 assesses depression, anxiety, and stress among individuals with seven items for each subscale. Its items are rated on a four-point Likert scale which ranges from 0 (*did not apply to me at all, never*) to 3 (*applied to me very much, or most of the time, almost always*). Participants' responses are added together for each subscale to get a total score (for each subscale) which ranges between 0 and 42 (scores for each subscale were doubled according to the scoring guideline) (29). The severity levels for depression were normal (0–9), mild (10–13), moderate (14–20), severe (21–27), and extremely severe (28 and above). The severity levels for anxiety were normal (0–7), mild (8, 9), moderate (10–14), severe (15–19), and extremely severe (20 and above). The severity levels for stress were normal (0–14), mild (15–18), moderate (19–25), severe (26–33), and extremely severe (34 and above). The higher the DASS scores, the higher the level of that corresponding subscale. The Chinese DASS-21 version has acceptable to excellent internal consistency (Cronbach's α = 0.83 for depression subscale, 0.80 for anxiety subscale, and 0.82 for stress subscale, and 0.95 for the total DASS-21 scale) (30, 31). In the present study, the Cronbach's alpha coefficient for depression is 0.90, anxiety is 0.85, and stress is 0.88.

#### Insomnia Severity Index

The healthcare workers' sleep problems (over the past 2 weeks) were assessed using the ISI developed by Bastien et al. (32). ISI is a seven-item self-report scale that is rated on a five-point Likert-type scale which ranges from 0 (*no problem*) to 4 (*very severe problem*). Participants' responses are added together to generate a total score which ranges from 0 to 28 with five sub-scores being 0–7 (absence of insomnia), 8–14 (sub-threshold insomnia), 15–21 (moderate insomnia/clinical insomnia), and 22–28 (severe insomnia/clinical insomnia) (25). The Chinese version has an acceptable internal consistency (Cronbach's α = 0.81) (33). The Cronbach's alpha coefficient for the present study is 0.89.

#### Impact of Event Scale-6

The healthcare workers' post-traumatic stress disorder (PTSD) problems (over the past 7 days) were assessed using the IES-6 developed by Hosey et al. (34). IES-6 is a six-item self-report scale that is rated on a five-point Likert-type scale which ranges from 0 (*not at all*) to 4 (extremely). Participants' responses are averaged together to generate a mean score. It has a diagnosable cut-off score of 1.75 (yielding 0.88 sensitivity and 0.85 specificity) with those above 1.75 deemed to having PTSD. It has acceptable reliability (Cronbach's α of 0.86 to 0.91 over time) and validity indices (34). The Chinese version with linguistic validity was used for this study. Because we translated the IES-6 and modified items to the COVID-19 event for this study, we performed confirmatory factor analysis (CFA) to establish its validity for this study. Hence, the Cronbach's alpha coefficient for the present study is 0.86, CFI 0.991, TLI is 0.985, RMSEA is 0.053, and SRMR is 0.054 (see Supplementary Table 1 for details).

#### Perceived Stigma Scale From COVID-19

The healthcare workers' perceived stigma from COVID-19 was assessed using the PSSC. Specifically, the present authors modified the perceived stigma scale developed by Williams et al. (35). Although Williams's et al. (35) perceived stigma scale originally focused on perceived stigma of racial difference, this scale has been revised for weight status and translated in Chinese with satisfactory psychometric properties (36, 37). In other words, the scale has the ability to assess other types of populations. Therefore, the present authors further revised the words of the items for the perceived stigma scale to focus on COVID-19 instead of the original perceived stigma using eight-item self-report scale that is rated on a binary response scale (Yes = 1 or No = 0). Participants' responses are added together to generate a total score which ranges from 0 to 8 with a higher score indicating higher levels of perceived stigma. The Chinese version has an acceptable internal consistency (Cronbach's α = 0.88). Because we modified the items to focus on COVID-19 for this study, we performed CFA to establish its validity for this study. Hence, the Cronbach's alpha coefficient for the present study is 0.82, CFI is 0.994, TLI is 0.991, RMSEA is 0.028, and SRMR is 0.074 (see Supplementary Table 1 for details).

#### Self-Stigma Scale From COVID-19

The healthcare workers' self-stigma from COVID-19 was assessed using the SSSC developed by the present authors. Specifically, the present authors modified the self-stigma scale developed by Mak and Cheung (38). Although Mak and Chueng's (38) self-stigma scale focused on self-stigma of mental illness and other minorities, this scale has been revised for specific learning disabilities (39) and substance use disorder (40–42). In other words, the scale has the ability to assess other types of populations. Therefore, the present authors further revised the words of the items for self-stigma scale to focus of COVID-19. The SSSC is a nine-item self-report scale that is rated on a four-point Likert-type scale which ranges from strongly disagree (1) to strongly agree (4). Participants' responses are averaged together to generate a mean score. It has a cut-off score of 2.5 which divides participants into having either low or high level of self-stigma. The Chinese version has an acceptable internal consistency (Cronbach's α = 0.89–0.95) (40–42). Because we modified the items to focus on COVID-19 for this study, we performed CFA to establish its validity for this study. Hence, the Cronbach's alpha coefficient for the present study is 0.92, CFI is 0.993, TLI is 0.991, RMSEA is 0.043, and SRMR is 0.059 (see Supplementary Table 1 for details).

### Data Analysis

Participants' demographic information was presented using descriptive statistics which included mean (SD) and frequency (percentage). Also, Pearson's *r* was used to examine the bivariate correlations among the variables of this study. Additionally, two hierarchical linear regression models were used to examine the factors that predict PTSD and insomnia among the participants. More specifically, the factors (i.e., perceived stigma, depression, anxiety, stress, fear of COVID-19, and self-stigma) that were significantly associated with PTSD or insomnia were entered in the regression models as potential predictors for PTSD or insomnia. Age and gender were controlled for in these models. We tested the multicollinearity of our variables and the results indicated that they were all below limits (43). All statistical analyses were conducted using SPSS version 22 software (IBM SPSS Statistics for Windows, Version 22.0. Armonk, NY: IBM Corp).

## Results

The participants (n = 500) in this study had a mean age of 32.96 (SD = 7.99) years with the majority being females (91.6%), college-educated (88.2%), and nurses (89%). Majority of the participants had not quarantined (92%) or not been tested for COVID-19 (80.6%) before the data collection (see Table 1).

**Table 1:** Participant characteristics (*n* = 500).

**Variable**	**Mean (SD) or *n* (%)**	**Missing *n***
Age	32.96 (7.99)	3
Gender		2
Male	40 (8%)	
Female	458 (91.6%)	
Education		4
Junior high	1 (0.2%)	
Senior high	16 (3.2%)	
College	441 (88.2%)	
Master	36 (7.2%)	
PhD	2 (0.4%)	
Occupation		18
Doctors	21 (4.2%)	
Nurses	445 (89%)	
Others	16 (3.2%)	
**Contact with COVID-19 patients**	
Yes, direct contact	271 (54.2%)	
Yes, indirect contact	140 (28%)	
Yes, intern with direct contact	48 (9.6%)	
Yes, intern with indirect contact	41 (8.2%)	
Monitor		30
Home quarantine after contact with COVID-19 cases	6 (1.2%)	
Home quarantine but no contact with COVID-19 cases	4 (0.8%)	
No quarantine	460 (92%)	
Number of COVID-19 tests		7
No	403 (80.6%)	
1	32 (6.4%)	
2	21 (4.2%)	
3 or more	37 (7.4%)	

Table 2 shows the prevalence levels of PTSD symptoms, insomnia, and psychological distress among participants. Specifically, it was observed that 15.4% of the participants had PTSD symptoms. A total of 44.6% had different levels of insomnia (6.4% for clinical insomnia) with the majority (38.2%) having a subthreshold level of insomnia. A total of 25.6% of participants had different levels of depressive symptoms with 17.4% having a moderate or above level of depression. A total of 30.6% of participants had different levels of anxiety symptoms with 23.6% having a moderate or above level of anxiety. A total of 23.4% of participants had various levels of stress with 15% having a moderate or above level of stress.

**Table 2:** Prevalence of post-traumatic stress disorder symptoms, insomnia, and psychological distress.

	**Percentage %**
Post-traumatic stress disorder symptoms	15.4
Insomnia	44.6
Subthreshold	38.2
Moderate	6.2
Severe	0.2
Depressive symptoms	25.6
Mild	8.2
Moderate	12
Severe	3.6
Extremely severe	1.8
Anxiety symptoms	30.6
Mild	7
Moderate	14
Severe	6
Extremely severe	3.6
Stress	23.4
Mild	8.4
Moderate	9.8
Severe	4
Extremely severe	1.2

Table 3 shows the interrelationship between perceived stigma, depressive symptoms, anxiety symptoms, stress, self-stigma, PTSD symptoms, insomnia, and fear of COVID-19. All the correlation coefficients (*r* = 0.127–0.838) were positive and significant (*ps* < 0.01) except for the relationship between self-stigma and PTSD symptoms which was not significant (*r* = 0.082, *p* = 0.065).

**Table 3:** Correlation matrix among studied variables.

		**1**	**2**	**3**	**4**	**5**	**6**	**7**	**8**
1	Perceived Stigma	–							
2	Depression	0.183^**^	–						
3	Anxiety	0.205^**^	0.831^**^	–					
4	Stress	0.175^**^	0.816^**^	0.838^**^	–				
5	Self-stigma	0.261^**^	0.166^**^	0.225^**^	0.239^**^	–			
6	Post-traumatic Stress Disorder	0.141^*^	0.307^**^	0.369^**^	0.318^**^	0.082	–		
7	Insomnia	0.127^*^	0.496^**^	0.547^**^	0.552^**^	0.193^**^	0.342^**^	–	
8	Fear of COVID-19	0.164^**^	0.274^**^	0.333^**^	0.313^**^	0.355^**^	0.444^**^	0.342^**^	–
	Mean	1.65	5.72	5.34	8.99	2.73	1.00	7.15	17.98
	SD	1.90	7.41	6.46	8.57	0.67	0.65	4.64	5.77

* *p < 0.01*;

** *p < 0.001*.

Table 4 shows the factors that predict PTSD symptoms and insomnia among the participants after adjusting for age and gender. In all, the factors predict about 27.2% of the factors needed for PTSD symptoms [*F*_(7, 487)_ = 25.999, *p* < 0.001] with anxiety symptoms [standardised coefficient (β) = 0.274, *p* = 0.001] and fear of COVID-19 (β = 0.386, *p* < 0.001) being the factors that significantly predict PTSD symptoms. Also, the factors used for insomnia predicted about 35.1% of all the factors needed for predicting insomnia [*F*_(8, 486)_ = 32.822, *p* < 0.001] with anxiety symptoms (β = 0.239, *p* = 0.002), stress (β = 0.283, *p* < 0.001), and fear of COVID-19 (β = 0.171, *p* < 0.001) being the factors that significantly predicted insomnia among the participants. Supplementary analysis for factors that predict PTSD symptoms and insomnia among doctors, nurses, and other healthcare workers (separately) revealed comparatively different findings (see Supplementary Tables 2–4).

**Table 4:** Predictive factors of post-traumatic stress disorder (PTSD) and Insomnia.

	**PTSD**				**Insomnia**			
	**B**	**SE**	***B***	***p*-value**	**B**	**SE**	***B***	***p*-value**
**Step 1**								
Constant	0.737	0.123	–	<0.001	5.954	0.880	–	<0.001
Age	0.008	0.004	0.096	0.033	0.037	0.026	0.065	0.150
Gender	0.110	0.106	0.046	0.301	−0.850	0.760	−0.050	0.264
**Step 2**								
Constant	−0.051	0.131	–	0.698	2.184	1.043	–	0.037
Age	0.004	0.003	0.045	0.248	−0.001	0.021	−0.002	0.954
Gender	0.314	0.094	0.133	0.001	0.342	0.642	0.020	0.594
Perceived stigma	0.007	0.013	0.022	0.579	−0.007	0.093	−0.003	0.944
Depression	−0.004	0.013	−0.020	0.792	0.018	0.091	0.014	0.845
Anxiety	0.055	0.016	0.274	0.001	0.342	0.111	0.239	0.002
Stress	−0.004	0.012	−0.024	0.756	0.305	0.079	0.283	<0.001
Fear of COVID-19	0.043	0.005	0.386	<0.001	0.137	0.033	0.171	<0.001
Self-stigma	–	–	–	–	0.007	0.032	0.009	0.818
**R**^**2**^ **(Adjusted** ***R**^**2**^***)**		27.2% (26.2%)				35.1% (34.0%)		
***ΔR**^**2**^*		26.1%				34.4%		
***ΔF***		34.863^***^				42.935^***^		

† *Age and gender were adjusted for the models*.

*** *p < 0.001*.

## Discussion

This study examined the prevalence of PTSD symptoms, sleep problems, psychological distress, and their correlates among healthcare workers in Tainan, Taiwan. In general, the findings on the prevalence rate of psychological problems among healthcare workers in Taiwan is a cause for concern. That is, about a fifth (15%) of the frontline healthcare workers may experience PTSD, a little below half (44.6%) of healthcare workers may experience sleep problems (6.4% clinically significant), about a fourth (25.6%) may experience depression, more than a fifth (23.4%) may experience stress, and a little below a third (30.6%) experiencing anxiety problems. Although these findings indicate symptoms of mental health conditions (i.e., PTSD and psychological distress), the results should be taken seriously and managed as they can lead to a complete mental health condition (12, 13). The root cause may be stress and fear of contracting COVID-19 as a frontline healthcare worker. These findings call for pre-emptive action in offering effective mental health services (e.g., relaxation therapy, desensitisation, grief coping) to healthcare workers especially COVID-19 frontline workers, to help cope with the challenges associated with COVID-19 (14, 44). These current findings are consistent with previous findings (17, 45, 46). In mainland China, the closest neighbour of Taiwan, a high prevalence of anxiety (53%), depression (56%), insomnia (79%), and PTSD (11%) was reported among medical workers (45). A systematic review and cumulated meta-analysis of studies on the psychological states of Chinese medical staff during COVID-19 revealed that the medical staff exhibited a substantial prevalence of anxiety symptoms (27%), depression symptoms (26.2%), stress-related symptoms (42.1%), and sleep problems (34.5%) (46). The findings of these studies support the current findings. Although Taiwan has fewer COVID-19 case reports than mainland China, the frontline healthcare workers' current prevalence rates of psychological distress may be a reflection of so many factors including fear of COVID-19 due to the global impact and their special relations to mainland China. That said, even in a safe country, caring for the mental health problems of the frontline healthcare workers is still important (47, 48). Furthermore, other studies reported that there is an increased rate of mental health problems among frontline workers (15, 16). Therefore, tackling the mental health issue is important for healthcare workers worldwide.

The significantly positive interrelationships between perceived stigma, depression, anxiety, stress, self-stigma, PTSD, insomnia, and fear of COVID-19 found in the Pearson correlations signify that as one of these variables increases, the other correlated variable also increases and vice versa. Hence, as frontline healthcare workers' fear of COVID-19 increases, their anxiety levels may also increase. These findings are supported by previous COVID-19 studies among other populations (4–6, 49). This may suggest that COVID-19 is significantly related to other psychological problems (e.g., hypochondriasis, internet, or social media addiction) that were not included in this study (50).

Further analysis indicated that anxiety symptoms and fear of COVID-19 were significant predictors of PTSD. That is, increased anxiety and fear of COVID-19 may lead to higher chances of PTSD. Thus, healthcare workers who show or experience considerable anxiety symptoms and fear of COVID-19 may have or going to have PTSD symptoms. Hence, there may be the need to examine and offer appropriate mental healthcare services (e.g., relaxation therapy, desensitisation, grief coping) to these individuals (especially those frontline workers who come into contact with COVID-19 patients) to help manage or prevent future PTSD. However, more factors may predict PTSD during this COVID-19 pandemic period as the factors used in this study accounted for 27.2% of all PTSD factors. Also, anxiety, stress, and fear of COVID-19 were found to predict insomnia among healthcare workers. Specifically, increased anxiety, stress, and fear of COVID-19 may lead to sleeping problems among healthcare workers. The transactional model of stress suggested that stress has the potential of triggering predisposed disorders which supports this finding (12). Furthermore, more factors may predict sleep problems during this COVID-19 pandemic period as the factors used in this study accounted for 35.1% of all possible factors for sleep problems (18, 51). In addition, anxiety and fear of COVID-19 seem to be central to PTSD and sleep problems which suggest that authorities should do their possible best to allay the fears of frontline healthcare workers about COVID-19 by providing what is needed for workers' safety on the job. That also suggests that adequate personal protective equipment, COVID-19-related training and treatment resources aside from COVID-19-related information should be made available to workers (especially those frontline workers who come into contact with COVID-19 patients) to ease their doubts and fears about COVID-19 (14, 44).

### Limitations

This study has some limitations. Firstly, a cross-sectional design was used which only provides associations between variables and so a longitudinal study may be needed to examine causality effects. Secondly, self-report measures were used which may be prone to social desirability bias although not expected in this study due to the robustness of the psychometric properties of the scales and the high ethical standards (ensured confidentiality and anonymity) with which the data was ascertained. Thirdly, different countries have different COVID-19 policies which may significantly affect the results hence, we recommend replications in other countries in order to give more comprehensive information on challenges among healthcare workers during COVID-19 pandemic. Fourthly, females and nurses as healthcare workers were overly represented in this study which may have influenced the results. Hence, this may limit the generalisation to other genders (e.g., males) and healthcare workers. Fifthly, as only questionnaires were used in this study, it is safe to assume that all the mental health conditions mentioned were at the symptomatic levels and should not be taken as a diagnosed disorder. Also, we did not collect data on participants' previous levels of psychological distress, insomnia (or psychiatric disorders) and other demographic characteristics such as work experience which could have enriched our results.

## Conclusion

The prevalence rate of psychological problems during COVID-19 pandemic reveals a worrisome view of the mental health challenges among healthcare workers in Taiwan. Anxiety and fear of COVID-19 as well as anxiety, stress, and fear of COVID-19 are the significant predicting factors for PTSD and sleep problems among healthcare workers respectively. It also suggests that healthcare services be open to all healthcare workers during the COVID-19 pandemic.

## Relevance for Clinical Practise

The findings from this study revealed that anxiety symptoms and fear of COVID-19 are the common predictive factors of PTSD and sleep problems among COVID-19 frontline healthcare workers. This implies that COVID-19 frontline healthcare workers may be manifesting symptoms of PTSD and sleep problems predictably due to increased anxiety symptoms and fear of COVID-19 (52). Hence, the mental health condition of frontline healthcare workers is important considering that they have to care for potential COVID-19 patients. It is, therefore, recommended that mental healthcare services be open to all healthcare workers during the COVID-19 pandemic.

## Data Availability Statement

The raw data supporting the conclusions of this article will be made available by the authors, without undue reservation.

## Ethics Statement

The studies involving human participants were reviewed and approved by Institute of Review Board of the National Cheng Kung University Hospital. The patients/participants provided their written informed consent to participate in this study.

## Author Contributions

M-YL, DA, CS, Y-JK, Y-PC, C-YL, P-LC, N-YK, and W-CK contributed to the conception and design of the study. M-YL, SK, and Y-HL organised the database. DA and C-YL performed the statistical analysis. M-YL, DA, SK, CS, and Y-HL interpreted the results. M-YL and DA wrote the first draught of the manuscript. C-YL wrote sections of the manuscript. CS, Y-HL, Y-JK, Y-PC, C-YL, P-LC, N-YK, and W-CK critically reviewed the manuscript. All authors contributed to manuscript revision, read and approved the submitted version.

## Conflict of Interest

The authors declare that the research was conducted in the absence of any commercial or financial relationships that could be construed as a potential conflict of interest.
