# Supplementary material for: The Prevalence of Post-traumatic Stress Disorder Symptoms, Sleep Problems, and Psychological Distress Among COVID-19 Frontline Healthcare Workers in Taiwan
Source: Front Psychiatry. 2021 Jul 12;12:705657. doi: 10.3389/fpsyt.2021.705657 (PMC8312888; doi:10.3389/fpsyt.2021.705657)
Supplement: Supplementary file 1 [file Data_Sheet_1.docx]

**Supplementary Analyses**

Table 1: Confirmatory factor analysis (CFA) fit indices of all the measures

|  | **Perceived Stigma Scale** | **Depression, Anxiety, Stress Scale** | **Self-Stigma Scale** | **Insomnia Severity Index** | **Fear of COVID-19 scale** | **Impact of Event Scale-6** |
| --- | --- | --- | --- | --- | --- | --- |
| χ2 | 27.680 | 144.870 | 52.370 | 55.700 | 57.310 | 21.640 |
| df | 20.000 | 186.000 | 27.000 | 14.000 | 14.000 | 9.000 |
| p-value | 0.120 | 0.990 | 0.002 | <0.001 | <0.001 | 0.010 |
| Comparative fit index | 0.994 | 1.000 | 0.993 | 0.980 | 0.981 | 0.991 |
| Tucker-Lewis index | 0.991 | 1.003 | 0.991 | 0.970 | 0.971 | 0.985 |
| RMSEA | 0.028 | 0.000 | 0.043 | 0.077 | 0.079 | 0.053 |
| 95% CI of RMSEA | 0.000, 0.051 | 0.000, 0.000 | 0.025, 0.061 | 0.057, 0.099 | 0.058, 0.100 | 0.024, 0.082 |
| SRMR | 0.074 | 0.053 | 0.059 | 0.075 | 0.070 | 0.054 |

CI = confidence interval; RMSEA = root mean square error of approximation; SRMR = standardized root mean square residual.

CFI > 0.9 indicates acceptable, TLI > 0.9 indicates acceptable, RMSEA < 0.08 indicates acceptable, and SRMR < 0.08 indicates acceptable.

Table 2: Predictive factors of Posttraumatic Stress Disorder (PTSD) and Insomnia among Medical Doctors†

|  | **PTSD** | | | |  | **Insomnia** | | | |
| --- | --- | --- | --- | --- | --- | --- | --- | --- | --- |
|  | B | SE | *B* | p-value |  | B | SE | *B* | p-value |
| Step 1 |  |  |  |  |  |  |  |  |  |
| Constant | 0.340 | 0.758 | ― | .659 |  | 0.811 | 5.116 | ― | .876 |
| Age | 0.025 | 0.017 | .333 | .152 |  | 0.168 | 0.112 | .336 | .149 |
| Gender | -0.080 | 0.347 | -.052 | .819 |  | -0.197 | 2.341 | -.019 | .934 |
| Step 2 |  |  |  |  |  |  |  |  |  |
| Constant | -1.712 | 1.130 | ― | .154 |  | 4.535 | 7.227 | ― | .542 |
| Age | 0.057 | 0.027 | .770 | .052 |  | 0.056 | 0.167 | .112 | .742 |
| Gender | 0.028 | 0.334 | .018 | .935 |  | -2.076 | 2.284 | -.198 | .381 |
| Perceived Stigma | -0.194 | 0.126 | -0.450 | .147 |  | 0.285 | 0.876 | 0.098 | .751 |
| Depression | 0.375 | 0.204 | 1.967 | .090 |  | -0.475 | 1.297 | -0.370 | .721 |
| Anxiety | -0.241 | 0.202 | -1.040 | .253 |  | 1.226 | 1.288 | 0.783 | .360 |
| Stress | -0.160 | 0.072 | -1.000 | .045 |  | 0.403 | 0.449 | 0.373 | .387 |
| Fear of COVID-19 | 0.093 | 0.033 | 0.699 | .014 |  | 0.090 | 0.205 | 0.101 | .667 |
| Self-stigma | ― | ― | ― | ― |  | -0.102 | 0.154 | -0.151 | .520 |
| **R^2^ (Adjusted R^2^)** | 54.2% (29.6%) | | | |  | 64.0% (40.1%) | | | |
| **ΔR^2^** | 42.6% | | | |  | 52.6% | | | |
| **ΔF** | 2.420 | | | |  | 2.926 | | | |

† Age and gender were adjusted for the models

Table 3: Predictive factors of Posttraumatic Stress Disorder (PTSD) and Insomnia among Nurses†

|  | **PTSD** | | | |  | **Insomnia** | | | |
| --- | --- | --- | --- | --- | --- | --- | --- | --- | --- |
|  | B | SE | *B* | p-value |  | B | SE | *B* | p-value |
| Step 1 |  |  |  |  |  |  |  |  |  |
| Constant | 1.145 | 0.321 | ― | .000 |  | 5.260 | 2.341 | ― | .025 |
| Age | 0.006 | 0.004 | .076 | .113 |  | 0.036 | 0.028 | .060 | .206 |
| Gender | -0.179 | 0.150 | -.057 | .235 |  | 0.418 | 1.096 | .018 | .703 |
| Step 2 |  |  |  |  |  |  |  |  |  |
| Constant | 0.675 | 0.286 | ― | .019 |  | 3.362 | 2.025 | ― | .098 |
| Age | 0.002 | 0.003 | .023 | .583 |  | -0.008 | 0.023 | -.013 | .743 |
| Gender | -0.349 | 0.132 | -.110 | .008 |  | -0.425 | 0.910 | -.018 | .641 |
| Perceived Stigma | 0.009 | 0.014 | 0.028 | .511 |  | 0.039 | 0.098 | 0.016 | .688 |
| Depression | -0.002 | 0.014 | -0.011 | .895 |  | 0.013 | 0.099 | 0.010 | .897 |
| Anxiety | 0.050 | 0.017 | 0.252 | .004 |  | 0.311 | 0.118 | 0.217 | .009 |
| Stress | 0.001 | 0.012 | 0.006 | .944 |  | 0.352 | 0.085 | 0.323 | <.001 |
| Fear of COVID-19 | 0.044 | 0.005 | 0.386 | <.001 |  | 0.123 | 0.035 | 0.151 | <.001 |
| Self-stigma | ― | ― | ― | ― |  | 0.006 | 0.034 | 0.008 | .850 |
| **R^2^ (Adjusted R^2^)** | 28.7% (27.5%) | | | |  | 36.1% (34.9%) | | | |
| **ΔR^2^** | 27.8% | | | |  | 35.7% | | | |
| **ΔF** | 33.692*** | | | |  | 40.164*** | | | |

† Age and gender were adjusted for the models

*** *p* <.001

Table 4: Predictive factors of Posttraumatic Stress Disorder (PTSD) and Insomnia among Other Professions†

|  | **PTSD** | | | |  | **Insomnia** | | | |
| --- | --- | --- | --- | --- | --- | --- | --- | --- | --- |
|  | B | SE | *B* | p-value |  | B | SE | *B* | p-value |
| Step 1 |  |  |  |  |  |  |  |  |  |
| Constant | 0.488 | 0.880 | ― | .589 |  | -1.570 | 5.180 | ― | .767 |
| Age | 0.016 | 0.021 | .223 | .462 |  | 0.091 | 0.123 | .208 | .472 |
| Gender | -0.023 | 0.448 | -.015 | .959 |  | 2.227 | 2.635 | .237 | .413 |
| Step 2 |  |  |  |  |  |  |  |  |  |
| Constant | -0.611 | 1.032 | ― | .570 |  | -2.974 | 7.751 | ― | .713 |
| Age | 0.025 | 0.026 | .350 | .372 |  | 0.113 | 0.196 | .257 | .583 |
| Gender | 0.067 | 0.409 | .044 | .875 |  | -0.310 | 3.508 | -.033 | .932 |
| Perceived Stigma | 0.190 | 0.116 | 0.539 | .140 |  | -0.842 | 0.889 | -0.386 | .375 |
| Depression | -0.104 | 0.072 | -0.676 | .185 |  | 0.572 | 0.584 | 0.601 | .360 |
| Anxiety | -0.315 | 0.197 | -1.124 | .149 |  | 0.556 | 1.475 | 0.320 | .718 |
| Stress | 0.280 | 0.118 | 1.728 | .045 |  | -1.045 | 0.882 | -1.043 | .275 |
| Fear of COVID-19 | 0.019 | 0.029 | 0.191 | .533 |  | -0.015 | 0.287 | -0.024 | .399 |
| Self-stigma | ― | ― | ― | ― |  | 0.320 | 0.356 | 0.500 | .961 |
| **R^2^ (Adjusted R^2^)** | 57.4% (20.2%) | | | |  | 45.7% (-16.4%) | | | |
| **ΔR^2^** | 52.7% | | | |  | 31.9% | | | |
| **ΔF** | 1.981 | | | |  | 0.685 | | | |

† Age and gender were adjusted for the models
